# Retraction: Optimum 3D Matrix Stiffness for Maintenance of Cancer Stem Cells Is Dependent on Tissue Origin of Cancer Cells

**DOI:** 10.1371/journal.pone.0350314

**Published:** 2026-05-28

**Authors:** The PLOS One Editors

After this article [1] was published, concerns were raised regarding results presented in Fig 1, and Figs 3-5.

Specifically:

- There appear to be repetitive areas within the Fig 1A 0.6 M Day 9 panel.
- There appear to be similarities between parts of the following panels:
  ◦ Figs 3D and 3E.
  ◦ Figs 3G and 3H.
  ◦ Figs 3I and 3J of this article, and Fig 5 14d 4T1-2.5 kPa in [2].
  ◦ Fig 3K of this article, Fig 5 5d 4T1-2.5 kPa in [2], Fig 5 5d 4T1-5.3 kPa in [2], Fig 5 8d 4T1-5.3 kPa in [2], and Fig 5 11d 4T1-2.5 kPa in [2].
- The Figs 4A and 4C panels appear more similar than expected from independent results.
- In Fig 5A, multiple panels show regions that appear discontinuous with the rest of the image directly surrounding these regions.

Regarding the above concerns for Figs 1A, 3D-3E, and 3G-3K, the corresponding author stated that the observed similar fluorescent features reflect expected underlying morphological uniformity of the cells and tumorspheres formed in the PEGDA matrix under controlled culture conditions and imaged using standardized fluorescence microscopy settings. Regarding the FACS panels in Figs 4A and 4C, the corresponding author stated that these plots share similar distribution patterns consistent with CD44 and CD24 expression under comparable culture conditions. The corresponding author provided incomplete image data underlying Figs 1 and 3-5. Upon editorial review, the data and responses provided did not resolve the above concerns.

In light of the above concerns that question the reliability and integrity of the reported results, the *PLOS One* Editors retract this article.

All authors did not agree with the retraction and stand by the article’s findings.

Figs 3I, 3J, and 3K report material similar to that published in [2], published in 2012 by Sage, under a restrictive license. These figures are not offered under a CC BY license and are therefore excluded from this article’s [1] license. Please see [2] for details regarding the licensing for the image material presented in Figs 3I, 3J, and 3K.
